# Perceived Electronic Health Record System Success and Its Associations With Healthcare Quality, Patient Safety, and Operational Efficiency in a Saudi Tertiary Hospital: A Cross-Sectional Survey Study

**DOI:** 10.7759/cureus.117458

**Published:** 2026-10-04

**Authors:** Yasser Alshahrani, Ahmed Hazazi, Tariq Falqi, Rufaidah Dabbagh

**Affiliations:** 1 Department of Public Health, Aseer Health Cluster, Abha, SAU; 2 Department of Public Health, College of Health Sciences, Prince Musaid Bin Abdulrahman University, Riyadh, SAU; 3 Southern Region Sector, Public Health Authority (Weqaya), Abha, SAU; 4 Department of Family and Community Medicine, College of Medicine, King Saud University, Riyadh, SAU; 5 Women’s Health Research Chair, Deanship of Scientific Research, King Saud University, Riyadh, SAU

**Keywords:** electronic health records, healthcare quality, information system success, operational efficiency, patient safety, saudi arabia, technical support, training

## Abstract

Background

Electronic health record (EHR) systems are widely implemented to support safer, higher-quality, and more efficient care. However, evidence regarding their perceived benefits in recently digitized tertiary hospitals remains limited. This study examined the associations of perceived EHR system success with perceived healthcare quality and patient safety and perceived operational efficiency. It also assessed the independent contribution of training and technical support and whether this support modified either association.

Methodology

A cross-sectional survey was conducted at Aseer Central Hospital, Saudi Arabia, from April 1 to 30, 2026. An expert-reviewed electronic questionnaire was administered to clinical and administrative employees using the VIDA EHR. Hierarchical linear regression models were fitted separately for perceived healthcare quality and patient safety and perceived operational efficiency, adjusting for demographic and professional characteristics. Training and technical support was examined as a main effect and through a prespecified interaction with perceived EHR system success.

Results

The analytic sample included 328 employees. Perceived EHR system success was positively associated with perceived healthcare quality and patient safety (B = 0.771, 95% confidence interval (CI) = 0.660 to 0.882; β = 0.711; p < 0.001) and perceived operational efficiency (B = 0.668, 95% CI = 0.532 to 0.804; β = 0.571; p < 0.001). The final models explained 65.6% and 55.5% of outcome variance, respectively. Training and technical support was associated with perceived operational efficiency (B = 0.214, 95% CI = 0.103 to 0.325; β = 0.199; p < 0.001), but not with perceived healthcare quality and patient safety in the primary model (p = 0.093). Neither interaction was statistically significant.

Conclusions

More favorable perceptions of EHR system success were associated with better perceived healthcare quality and patient safety and greater perceived operational efficiency. Training and technical support was independently associated with perceived operational efficiency but did not modify either association. Because the study was cross-sectional and used employee-reported perceptions, the findings should not be interpreted as causal effects or objective improvements in care.

## Introduction

Electronic health record (EHR) systems are central to contemporary health system strategies. The World Health Organization identifies digital health as an enabling component of health system strengthening [1]. The DeLone-McLean Information Systems Success Model explains how system quality, information quality, service quality, use, and user satisfaction contribute to net benefits [2]. Donabedian’s Structure-Process-Outcome framework complements this perspective by treating digital infrastructure as a structural input that can shape care processes and outcomes [3]. Together, these models support evaluation of both the functioning of an EHR and its perceived clinical and operational consequences.

In this study, perceived healthcare quality and patient safety refer to perceived improvements in documentation accuracy, continuity of care, communication, guideline adherence, and safety. Perceived operational efficiency refers to perceived reductions in duplicated work, time savings, workflow improvement, resource use, and operational cost efficiency. Previous research has associated EHR implementation with improved guideline adherence, documentation, and medication safety, although findings vary across settings and implementation maturity [4-6]. Training, usability, technical competence, and responsive support are repeatedly identified as conditions that may shape EHR-related benefits [7-9]. These findings suggest that EHR outcomes should be interpreted within their organizational context rather than attributed to technology alone.

Saudi Arabia provides a relevant setting in which to examine these relationships because EHR implementation is expanding alongside national health system transformation [10]. Earlier Saudi research described variable adoption and barriers involving computer literacy, training, and technical support [11], while multicenter evidence found generally favorable staff perceptions alongside persistent implementation concerns [12]. Aseer Health Cluster is a large regional health network serving southern Saudi Arabia [13]. A recent systematic review indicates that the efficiency and patient outcome effects of EHR implementation vary across organizational contexts [14]. Evidence from recently digitized tertiary hospitals outside the most frequently studied metropolitan Saudi settings remains limited.

Approximately one year after VIDA EHR deployment, this study applied the DeLone-McLean and Donabedian frameworks to evaluate perceived EHR system success and two perceived net benefit domains among employees of a tertiary referral hospital. Training and technical support was examined both as an independent organizational input and as a potential statistical modifier of the associations between EHR system success and the two outcomes.

The objective of this study was to examine the association of perceived EHR system success with two primary outcomes among employees at Aseer Central Hospital: (1) perceived healthcare quality and patient safety and (2) perceived operational efficiency. Perceived EHR system success was summarized descriptively on its original 1-5 scale; no a priori cutoff was used to classify scores as moderate or high. We hypothesized that higher perceived EHR system success scores would be positively associated with both outcomes. Training and technical support was also examined as an independent predictor and as a potential effect modifier of the association between perceived EHR system success and each outcome.

## Materials and methods

Study design

A cross-sectional observational design was used to examine associations among perceived EHR system success, perceived healthcare quality and patient safety, perceived operational efficiency, and training and technical support among healthcare employees at Aseer Central Hospital (ACH). ACH was selected because it is the principal adult tertiary referral hospital in the Aseer region and had implemented a hospital-wide EHR approximately one year before data collection, providing an appropriate setting to evaluate early post-implementation perceptions across diverse clinical and administrative departments. Reporting followed the STROBE statement for cross-sectional studies [15].

Setting and participants

The study was conducted at ACH, an adult tertiary referral hospital under the Saudi Ministry of Health in Abha, southern Saudi Arabia. ACH serves as a major referral center for the Aseer region, whose population was approximately 2.02 million in the 2022 national census [16]. The hospital does not provide pediatric or obstetric and gynecological services; these populations are served by a separate maternity and children’s hospital in Abha.

ACH adopted the VIDA EHR system in 2025 as part of the Saudi Vision 2030 Health Sector Transformation Program [10]. VIDA is a cloud-based, paperless EHR platform supplied by Cloud Solutions Co [17].

Eligible participants were healthcare employees actively using VIDA in their clinical or administrative duties, with at least three months of cumulative experience using any EHR system and active hospital service during the data collection period. All professional categories were eligible, including physicians, nurses, pharmacists, allied health professionals, and administrative staff.

Recruitment and sampling

A nonprobability convenience sampling strategy was used [18]. Invitations were distributed through official hospital WhatsApp staff groups, supervisor announcements during shift handovers and departmental meetings, and posters containing a QR code linked to the questionnaire. The electronic invitation could be forwarded by recipients. For an estimated eligible workforce of 1,500 to 2,000 employees, a conservative finite population calculation assuming 50% response variability, 95% confidence, and a 5% margin of error indicated a required sample of approximately 306 to 323 participants. This calculation was intended to support the precision of the survey estimates and was not a formal power calculation for the hierarchical regression models or interaction tests. The final analytic sample included 328 participants.

Recruitment was open from April 1 to 30, 2026. Because all three recruitment channels allowed the invitation to be shared, the number of employees who received an invitation could not be determined, and a formal response rate was not calculated.

Instrument

Data were collected using a structured questionnaire developed for this study. An initial item pool was reviewed by a multidisciplinary panel with expertise in clinical practice, health informatics, and academic research; revisions were made to strengthen face and content validity. The refined questionnaire was piloted with 17 healthcare employees at ACH, who were excluded from the main analysis. It contained demographic and professional characteristics and four study-specific multi-item composites.

The four composites and their items were defined a priori based on the conceptual content of the items and their alignment with the DeLone-McLean and Donabedian frameworks. The proposed grouping was reviewed by the multidisciplinary expert panel and retained after pilot testing. The composites were not derived or validated using exploratory or confirmatory factor analysis.

All substantive items used a five-point Likert response format [19], ranging from 1 (strongly disagree) to 5 (strongly agree). The composites were perceived EHR system success (six items), perceived healthcare quality and patient safety (five items), perceived operational efficiency (five items), and training and technical support (four items). Each score was calculated as the arithmetic mean of its constituent items on the original 1-5 scale. The complete wording of all 20 substantive items, their response scale, and their assignment to the four composites are provided in Appendix 1. The questionnaire was administered in English, the working language of the VIDA interface and clinical documentation at ACH.

Data collection

The questionnaire was administered through Google Forms from April 1 to 30, 2026. Substantive items were configured as mandatory; nevertheless, 26 exported records (7.1% of the 366 returned records) contained missing information required for the planned composite score or regression analyses. These records were excluded without imputation using complete case analysis. Because the deidentified export did not retain the technical information needed to determine why records remained incomplete despite the mandatory response settings, the mechanism of incompleteness could not be established. Two reminders were distributed, at the end of week one and approximately 48 hours before closure. No personal identifiers were collected, and the de-identified export was stored in a password-protected account accessible only to the research team.

Data analysis

Analyses were performed using SPSS Statistics version 31 (IBM Corp., Armonk, NY, USA). Internal consistency was assessed using Cronbach’s alpha [20,21], with values of 0.70 or higher considered acceptable. Composite distributions were summarized using means, standard deviations, medians, interquartile ranges, skewness, and kurtosis. Pearson correlations were examined descriptively before regression.

Hierarchical linear regression was fitted separately for perceived healthcare quality and patient safety and perceived operational efficiency. Block 1 included gender, age group, professional role, department, years of clinical experience, and years using an EHR. Professional role and department were dummy-coded, with physician and medical unit as the reference categories. Ordered categories for age group, years of clinical experience, and years using an EHR were entered as ordinal terms in the primary models to preserve their ordering and limit the number of model parameters. This coding assumes an approximately linear change across successive categories. Therefore, an additional sensitivity analysis entered these variables as categorical indicator variables, with the lowest category used as the reference. Block 2 added mean-centered perceived EHR system success and training and technical support. Based on prior evidence that implementation support may condition EHR-related benefits [7-9,22], Block 3 added their multiplicative interaction. This interaction analysis was prespecified. Simple slopes at 1 SD above and below the support score mean were planned only if the interaction reached p < 0.05.

The composite outcomes were analyzed as approximately continuous variables because each represented the mean of five Likert items, and modeling the original 1-5 scales allowed direct interpretation of the regression coefficients. The models using the original outcome scales were considered the prespecified primary analyses.

Model diagnostics included variance inflation factor (VIF) and tolerance values to assess multicollinearity, visual review of residual plots, and Cook’s distance to assess potentially influential observations. The final models included 21 predictor terms. Unstandardized coefficients, standard errors, standardized coefficients, 95% confidence intervals (CIs), and two-sided p-values were reported, with p < 0.05 indicating statistical significance.

Invariant responding was defined as selecting the same response option across all 20 substantive questionnaire items. These respondents were retained in the primary analysis because this response pattern alone did not establish that an individual response was invalid. The first sensitivity analysis excluded the 48 respondents (14.6% of the full sample) with invariant response patterns and refitted both models. Because both outcomes were negatively skewed, the second sensitivity analysis refitted the fully adjusted models after reflected-log transformation of each outcome using -ln(6 - y). The transformed outcome models were used to assess the robustness of the findings to outcome distribution and were not treated as replacements for the primary models. The third sensitivity analysis entered age group, years of clinical experience, and years using an EHR as categorical indicator variables rather than ordinal terms.

Ethical considerations

The study was conducted in compliance with the World Medical Association Declaration of Helsinki [23] and the Implementing Regulations of the Law of Ethics of Research on Living Creatures issued by the National Committee of Bioethics, Saudi Arabia [24]. Ethical approval was obtained from the Aseer Institutional Review Board before recruitment (IRB-063-2026; April 1, 2026). The approved study title was “The Impact of Electronic Health Records (EHR) on Healthcare Quality and Cost Efficiency at Aseer Central Hospital.” Study information and informed consent were presented electronically before the questionnaire. Participation was voluntary, and no compensation was provided. No names, employee identifiers, email addresses, or other direct identifiers were collected. The de-identified data were stored in a password-protected account accessible only to the research team.

## Results

Description of participants

A total of 366 records were returned. In total, 12 (3.3%) respondents declined consent and did not proceed to the substantive questionnaire. A further 26 (7.1%) records contained missing information required for the planned composite score or regression analyses and were excluded without imputation using complete case analysis. The technical reason for these incomplete records could not be determined from the de-identified export. The final analytic sample comprised 328 employees (89.6% of returned records). This percentage is not a response rate because the number of employees who received the invitation was unknown.

The sample was approximately balanced by gender, with 178 (54.3%) male participants and 150 (45.7%) female participants, and was concentrated in the 30-39-year age group, which included 206 (62.8%) participants. Nurses comprised 94 (28.7%) participants, and physicians comprised 87 (26.5%) participants. Medical and surgical units together accounted for 146 (44.5%) respondents. Overall, 273 (83.2%) participants had at least five years of clinical experience, and 212 (64.6%) participants reported more than three years of cumulative EHR experience (Table 1).

**Table 1:** Sociodemographic and professional characteristics of the analytic sample (n = 328). Note: n = 328. Data are presented as n (%). Percentages may not sum to 100 because of rounding. EHR = electronic health record; ICU = intensive care unit

Characteristic	n (%)
Gender	
Male	178 (54.3)
Female	150 (45.7)
Age group	
<30 years	40 (12.2)
30–39 years	206 (62.8)
40–49 years	60 (18.3)
≥50 years	22 (6.7)
Professional role	
Physician	87 (26.5)
Nurse	94 (28.7)
Pharmacist	23 (7.0)
Allied health	68 (20.7)
Administrative	30 (9.1)
Other	26 (7.9)
Department/Unit	
Medical	76 (23.2)
Surgical	70 (21.3)
Emergency	25 (7.6)
ICU	17 (5.2)
Radiology	11 (3.4)
Laboratory	20 (6.1)
Pharmacy	23 (7.0)
Outpatient	19 (5.8)
Administrative	30 (9.1)
Other	37 (11.3)
Years of clinical experience	
<5 years	55 (16.8)
5–10 years	133 (40.5)
>10 years	140 (42.7)
Years using EHR	
<1 year	46 (14.0)
1–3 years	70 (21.3)
>3 years	212 (64.6)

Internal consistency of study composites

All four study-specific composites demonstrated acceptable internal consistency: perceived EHR system success (six items), Cronbach’s alpha = 0.889; perceived healthcare quality and patient safety (five items), Cronbach’s alpha = 0.932; perceived operational efficiency (five items), Cronbach’s alpha = 0.927; and training and technical support (four items), Cronbach’s alpha = 0.838. No item deletion results supported removing an item.

Distribution of study measures

Perceived EHR system success had a mean of 4.11 (SD = 0.90), a median of 4.33, and an interquartile range from 3.67 to 4.83. Perceived healthcare quality and patient safety had a mean of 4.19 (SD = 0.98) and a median of 4.60, while perceived operational efficiency had a mean of 4.03 (SD = 1.06) and a median of 4.40. Training and technical support had a mean of 3.74 (SD = 0.99) and a median of 4.00. The first three composites were negatively skewed, while training and technical support was approximately symmetric (Table 2). Item-level descriptive statistics and the complete wording of each item are reported in Appendix 1.

**Table 2:** Descriptive statistics for the four composite scores (n = 328). *Note.* n = 328. Data are presented as mean (M), standard deviation (SD), median (Mdn), first quartile (Q1), third quartile (Q3), skewness, and kurtosis. Composite scores are means of constituent five-point Likert items scored from 1 (strongly disagree) to 5 (strongly agree). Skewness and kurtosis are Fisher’s adjusted statistics (G1 and excess G2). EHR = electronic health record

Composite	Mean	SD	Median	Q1	Q3	Skewness	Kurtosis
Perceived EHR system success	4.112	0.904	4.33	3.67	4.83	−1.30	+1.39
Perceived healthcare quality and patient safety	4.191	0.981	4.60	4.00	5.00	−1.56	+1.99
Perceived operational efficiency	4.030	1.057	4.40	3.40	5.00	−1.12	+0.54
Training and technical support	3.736	0.987	4.00	3.00	4.50	−0.43	−0.69

Correlations among study measures

Pearson product-moment correlations among the four composite scores are reported in Table 3. All six pairwise correlations were positive and statistically significant at p < 0.001, with magnitudes ranging from r = 0.545 (perceived healthcare quality and patient safety with training and technical support) to r = 0.852 (perceived healthcare quality and patient safety with perceived operational efficiency). Perceived EHR system success was strongly correlated with perceived healthcare quality and patient safety (r = 0.789) and perceived operational efficiency (r = 0.694), and moderately correlated with training and technical support (r = 0.617). The pattern of associations is consistent with the DeLone-McLean and Donabedian frameworks and motivates the multivariable analyses that follow.

**Table 3:** Pearson product moment correlations among the four composite scores (n = 328). Note. n = 328. Values are Pearson product moment correlation coefficients (r). All p values are two-sided, and p < 0.05 was considered statistically significant. *** p < 0.001. EHR = electronic health record

Variable	1	2	3	4
1. Perceived EHR system success	—			
2. Perceived healthcare quality and patient safety	0.789***	—		
3. Perceived operational efficiency	0.694***	0.852***	—	
4. Training and technical support	0.617***	0.545***	0.552***	—

Adjusted associations with perceived healthcare quality and patient safety and perceived operational efficiency

Final block coefficients are presented in Table 4. Hierarchical linear regression models for perceived healthcare quality and patient safety and perceived operational efficiency are summarized in Table 5.

**Table 4:** Final block coefficients for both hierarchical regression models (n = 328). Note. n = 328. A p value < 0.05 was considered statistically significant. Reference categories were male for gender, physician for professional role, and medical for department. An em dash indicates that β is not applicable to the intercept. ICU = intensive care unit; EHR = electronic health record; B = unstandardized coefficient; CI = confidence interval; SE = standard error; β = standardized coefficient; p = two-sided p value

	Model 1: Perceived healthcare quality and patient safety				Model 2: Perceived operational efficiency			
Predictor	B (95% CI)	SE	β	P-value	B (95% CI)	SE	β	P-value
(Intercept)	4.494 (4.093 to 4.895)	0.204	—	<0.001	4.367 (3.875 to 4.859)	0.250	—	<0.001
Gender	0.028 (−0.110 to 0.167)	0.071	0.014	0.687	0.120 (−0.050 to 0.290)	0.087	0.057	0.167
Age group	−0.090 (−0.210 to 0.030)	0.061	−0.067	0.139	−0.033 (−0.180 to 0.114)	0.075	−0.023	0.663
Years of clinical experience	0.007 (−0.119 to 0.133)	0.064	0.005	0.911	−0.015 (−0.170 to 0.139)	0.078	−0.011	0.845
Years using EHR	−0.057 (−0.162 to 0.048)	0.054	−0.042	0.289	−0.145 (−0.275 to −0.016)	0.066	−0.100	0.028
Role: Nurse	0.027 (−0.154 to 0.209)	0.092	0.013	0.766	−0.025 (−0.247 to 0.197)	0.113	−0.011	0.823
Role: Pharmacist	0.164 (−0.171 to 0.500)	0.171	0.043	0.336	0.270 (−0.142 to 0.681)	0.209	0.065	0.198
Role: Allied health	−0.164 (−0.386 to 0.059)	0.113	−0.068	0.149	−0.032 (−0.305 to 0.241)	0.139	−0.012	0.816
Role: Administrative	−0.121 (−0.393 to 0.150)	0.138	−0.036	0.380	−0.056 (−0.389 to 0.277)	0.169	−0.015	0.740
Role: Other	0.214 (−0.064 to 0.493)	0.142	0.059	0.131	0.261 (−0.081 to 0.602)	0.174	0.067	0.134
Department: Surgical	−0.047 (−0.247 to 0.154)	0.102	−0.020	0.647	0.021 (−0.225 to 0.267)	0.125	0.008	0.867
Department: Emergency	−0.114 (−0.395 to 0.166)	0.143	−0.031	0.423	−0.193 (−0.537 to 0.150)	0.175	−0.049	0.269
Department: ICU	−0.222 (−0.549 to 0.105)	0.166	−0.050	0.183	−0.695 (−1.096 to −0.295)	0.204	−0.146	<0.001
Department: Radiology	−0.025 (−0.414 to 0.365)	0.198	−0.005	0.901	0.170 (−0.308 to 0.647)	0.243	0.029	0.485
Department: Laboratory	0.088 (−0.229 to 0.404)	0.161	0.021	0.585	−0.138 (−0.525 to 0.250)	0.197	−0.031	0.485
Department: Pharmacy	0.009 (−0.320 to 0.338)	0.167	0.002	0.958	−0.068 (−0.471 to 0.335)	0.205	−0.017	0.739
Department: Outpatient	0.184 (−0.126 to 0.495)	0.158	0.044	0.244	0.039 (−0.342 to 0.419)	0.193	0.009	0.842
Department: Administrative	0.090 (−0.189 to 0.370)	0.142	0.027	0.525	0.023 (−0.319 to 0.365)	0.174	0.006	0.895
Department: Other	0.192 (−0.060 to 0.444)	0.128	0.062	0.135	0.103 (−0.205 to 0.412)	0.157	0.031	0.510
Perceived EHR system success (centered)	0.771 (0.660 to 0.882)	0.056	0.711	<0.001	0.668 (0.532 to 0.804)	0.069	0.571	<0.001
Training and technical support (centered)	0.078 (−0.013 to 0.168)	0.046	0.078	0.093	0.214 (0.103 to 0.325)	0.056	0.199	<0.001
EHR success x training/support	−0.048 (−0.116 to 0.019)	0.034	−0.057	0.162	−0.043 (−0.126 to 0.040)	0.042	−0.047	0.309

**Table 5:** Hierarchical regression model summaries for both dependent variables (n = 328). Note. n = 328. A p value < 0.05 was considered statistically significant. k = cumulative number of predictors; R² = coefficient of determination; Adj R² = adjusted coefficient of determination; ΔR² = change in R²; F change = F statistic for the change in model fit; df1 = numerator degrees of freedom; df2 = denominator degrees of freedom; df change = numerator degrees of freedom for the change; p = two-sided p value

Model	k	R²	Adj R²	F	df1	df2	P-value	ΔR²	F change	df change	P-value change
Model 1: Perceived healthcare quality and patient safety											
Model 1 Block 1 Covariates	18	0.105	0.053	2.01	18	309	0.009	-	-	-	-
Model 1 Block 2 Main effects	20	0.653	0.631	28.93	20	307	<0.001	+0.549	242.92	2	<0.001
Model 1 Block 3 Interaction	21	0.656	0.632	27.73	21	306	<0.001	+0.002	1.96	1	0.162
Model 2: Perceived operational efficiency											
Model 2 Block 1 Covariates	18	0.080	0.027	1.50	18	309	0.088	-	-	-	-
Model 2 Block 2 Main effects	20	0.553	0.524	19.02	20	307	<0.001	+0.473	162.56	2	<0.001
Model 2 Block 3 Interaction	21	0.555	0.524	18.16	21	306	<0.001	+0.002	1.04	1	0.309

For perceived healthcare quality and patient safety, Block 1 explained 10.5% of the variance (R² = 0.105; p = 0.009). Block 2 increased the explained variance by 54.9 percentage points (p < 0.001), and Block 3 produced a final R² of 0.656. In the final model, perceived EHR system success was positively associated with the outcome (B = 0.771, 95% CI = 0.660 to 0.882; β = 0.711; p < 0.001). Training and technical support was not independently significant (B = 0.078, 95% CI = -0.013 to 0.168; β = 0.078; p = 0.093). For perceived operational efficiency, Block 1 explained 8.0% of the variance (R² = 0.080; p = 0.088). Block 2 increased the explained variance by 47.3 percentage points (p < 0.001), and Block 3 produced a final R² of 0.555. Perceived EHR system success was positively associated with perceived operational efficiency (B = 0.668, 95% CI = 0.532 to 0.804; β = 0.571; p < 0.001), as was training and technical support (B = 0.214, 95% CI = 0.103 to 0.325; β = 0.199; p < 0.001). Intensive care unit (ICU) membership was associated with lower perceived operational efficiency (B = -0.695, 95% CI = -1.096 to -0.295; β = -0.146; p < 0.001), and years using an EHR showed a small negative association (B = -0.145, 95% CI = -0.275 to -0.016; β = -0.100; p = 0.028).

The final models included 21 predictor terms among 328 participants, providing approximately 16 observations per predictor term. The maximum VIF was 2.40, and the minimum tolerance was 0.417, indicating no important multicollinearity. Residual plots were reviewed, and departures related to the negatively skewed outcomes were further examined in the transformed outcome sensitivity analyses. The maximum Cook’s distance was 0.070 for the perceived healthcare quality and patient safety model and 0.048 for the perceived operational efficiency model. All values were below 1, suggesting that no single observation had an undue influence on either model.

Interaction between perceived electronic health record system success and training and technical support

The prespecified interaction between perceived EHR system success and training and technical support was not statistically significant for perceived healthcare quality and patient safety (B = −0.048, 95% CI = −0.116 to 0.019; β = −0.057; p = 0.162) or perceived operational efficiency (B = −0.043, 95% CI = −0.126 to 0.040; β = −0.047; p = 0.309). The increment in explained variance was ΔR² = 0.002 for both outcomes; therefore, the planned simple slopes analyses were not performed. The interaction coefficients are presented in Table 4, and the corresponding model change statistics are summarized in Table 5.

Sensitivity analysis

Three sensitivity analyses were conducted. First, 48 respondents (14.6% of the full sample) with invariant responses across all 20 substantive items were excluded, yielding 280 respondents (85.4% of the full sample). Perceived EHR system success remained associated with perceived healthcare quality and patient safety (B = 0.755, 95% CI = 0.628 to 0.882; β = 0.674; p < 0.001) and perceived operational efficiency (B = 0.644, 95% CI = 0.489 to 0.799; β = 0.530; p < 0.001). Training and technical support remained associated with perceived operational efficiency (B = 0.202, 95% CI = 0.072 to 0.333; β = 0.177; p = 0.003), and both interactions remained nonsignificant.

In the reflected-log models, perceived EHR system success remained significantly associated with both outcomes, and both interaction terms remained nonsignificant. Training and technical support was significantly associated with the transformed perceived healthcare quality and patient safety outcome (β = 0.162; p < 0.001), although it was not significant in the primary model using the original outcome scale (β = 0.078; p = 0.093). The transformation changed the distribution and scale of the outcome by reducing the influence of responses clustered near the upper end. Therefore, this association was considered sensitive to outcome specification. The model using the original outcome scale remained the prespecified primary analysis. Details are provided in Appendix 2.

In the additional sensitivity analysis using categorical indicator coding for age group, years of clinical experience, and years using an EHR, the focal findings remained substantively unchanged. Perceived EHR system success remained associated with perceived healthcare quality and patient safety (B = 0.769; p < 0.001) and perceived operational efficiency (B = 0.661; p < 0.001). Training and technical support remained nonsignificant for perceived healthcare quality and patient safety (B = 0.078; p = 0.095) and significant for perceived operational efficiency (B = 0.212; p < 0.001). Both interaction terms remained nonsignificant (p = 0.154 and p = 0.271, respectively). Details are provided in Appendix 2.

## Discussion

This study found generally favorable perceptions of EHR system success approximately one year after implementation. Higher perceived EHR system success was strongly associated with better perceived healthcare quality and patient safety and with greater perceived operational efficiency after adjustment for demographic and professional characteristics. Training and technical support was independently associated with perceived operational efficiency, but not with perceived healthcare quality and patient safety in the primary model, and did not significantly modify either association.

The positive association with perceived healthcare quality and patient safety is consistent with evidence linking well-implemented EHR systems to improved documentation, guideline adherence, continuity, and medication safety [4-6]. The magnitude observed here was comparatively large. Shared source measurement and conceptual overlap between the assessments of EHR functioning and its perceived benefits may have inflated the association [25]. The estimate should therefore be interpreted as an association between employee perceptions, not as evidence that the EHR caused objective improvements in care quality or safety.

The association with perceived operational efficiency similarly accords with research suggesting that EHR benefits depend on workflow integration, usability, and organizational implementation [5,7-9]. Training and technical support remained independently associated with perceived operational efficiency, suggesting that accessible support may reduce workflow disruption and help employees use system functions more effectively. Because one support item measured belief in the value of continuing training rather than training actually received, the composite is best interpreted as a broader indicator of training and support perceptions.

Training and technical support was not significantly associated with perceived healthcare quality and patient safety in the prespecified primary analysis. Although the association became significant after reflected-log transformation, the transformation changed the distribution and scale of the outcome. This difference indicates that the association was sensitive to outcome specification. Therefore, the nonsignificant result from the primary model remains the main finding, while the transformed result should be interpreted cautiously as a secondary finding.

The nonsignificant interactions indicate that the slopes relating perceived EHR system success to the two outcomes did not differ detectably across levels of reported training and technical support in this sample. This does not establish equivalence or rule out effect modification in other populations. Ceiling-leaning distributions and the greater sample size requirements of interaction tests may have reduced sensitivity to small conditional effects [22].

The findings add evidence from Aseer to the growing Saudi literature on EHR implementation [11,12,14,26]. Lower perceived operational efficiency among ICU staff and the negative association between years of EHR use and perceived operational efficiency were adjusted secondary findings. Documentation intensity and time-sensitive workflows in the ICU, and greater awareness of system limitations among employees with longer EHR experience, are possible explanations. However, these mechanisms were not directly measured or tested in this study and should therefore be regarded as hypotheses rather than established explanations. These findings require replication in other settings using measures specifically designed to examine workflow demands, user experience, and objective operational outcomes.

Strengths and limitations

To our knowledge, this is the first study conducted at ACH, and among the first in the Aseer region, to examine perceived EHR system success, perceived healthcare quality and patient safety, perceived operational efficiency, and training and technical support within a single adjusted analytical framework. Additional strengths include multidisciplinary expert review and pilot testing of the questionnaire, inclusion of diverse clinical and administrative roles, adjustment for major demographic and professional characteristics, prespecified interaction testing, and three sensitivity analyses. The final sample also exceeded the estimated minimum required under the study planning assumptions.

Several limitations should be considered. The cross-sectional design cannot establish temporal direction or causality. All substantive variables were collected using a single self-administered questionnaire, creating potential common method bias [25] and conceptual overlap between the exposure and outcomes. The study-specific composites demonstrated good internal consistency; however, their dimensionality and construct validity were not evaluated using exploratory or confirmatory factor analysis. Internal consistency alone does not confirm that each composite represents a distinct construct. In particular, the high correlation between perceived healthcare quality and patient safety and perceived operational efficiency (r = 0.852) indicates substantial empirical overlap between the two outcomes. Their discriminant validity therefore cannot be confirmed, and the results should not be interpreted as demonstrating effects on fully independent outcome domains. Further validation in independent and more diverse samples is needed. The training and technical support composite also combined support-related perceptions with a belief about continuing training. Its association with perceived healthcare quality and patient safety changed in statistical significance after outcome transformation, indicating that this secondary finding was sensitive to outcome specification and should be interpreted cautiously.

The original sample size calculation was based on the precision of a finite-population survey estimate and was not a formal power calculation for the hierarchical regression models or interaction tests. Although the final models included approximately 16 observations per predictor term, the study may still have had limited power to detect small interaction effects. Age group, years of clinical experience, and years using an EHR were entered as ordered terms in the primary models, which assumes an approximately linear change across categories; however, the focal findings remained substantively unchanged when these variables were entered as categorical indicator variables. The 48 invariant response records (14.6%) may indicate limited engagement or a response quality concern, but a uniform pattern alone did not establish that an individual response was invalid. The principal conclusions remained unchanged when these records were excluded.

Convenience recruitment and the forwardable survey link may have favored employees with stronger opinions or greater engagement with the EHR, and the number invited was unknown. The anonymous design prevented assessment of duplicate participation. Twenty-six unexpectedly incomplete records (7.1% of the 366 returned records) were excluded, and the mechanism of incompleteness could not be determined. English-only administration may have reduced participation or response accuracy among employees less comfortable with English. Finally, the single-hospital setting, use of one EHR product, and assessment approximately one year after implementation limit generalizability to other hospitals, care levels, products, and implementation stages.

Implications and future research

The findings have practical implications for hospitals implementing or optimizing EHR systems. Technical deployment should be accompanied by accessible, role-specific training, responsive user support, and continued evaluation of how the system fits clinical and administrative workflows [7-9]. Particular attention may be warranted in high-acuity settings, such as ICUs, where workflow demands and documentation requirements may differ from those in other departments. However, because the present study was cross-sectional and based on staff perceptions, these findings should inform organizational assessment and improvement efforts rather than be interpreted as evidence of causal effects.

Future studies should use multicenter and longitudinal designs to examine how perceptions and outcomes change over time and across healthcare settings. Research should also incorporate validated multidimensional measures and objective indicators of patient safety, healthcare quality, and operational efficiency. Probability-based sampling, where feasible, could improve representativeness. Future questionnaires should distinguish between training received, availability of technical support, satisfaction with support, and beliefs about the value of continued training. This distinction may help clarify which organizational support mechanisms are most strongly associated with successful EHR use.

## Conclusions

More favorable perceptions of EHR system success were strongly associated with higher perceived healthcare quality and patient safety and with greater perceived operational efficiency among staff at ACH. Training and technical support was independently associated with perceived operational efficiency, but it did not significantly modify the associations between perceived EHR system success and either outcome. In the prespecified primary analysis, training and technical support was not significantly associated with perceived healthcare quality and patient safety; the significant result after outcome transformation was a secondary sensitivity finding and should be interpreted cautiously. These findings represent associations among employee-reported perceptions and should not be interpreted as evidence that the EHR itself caused improvements in objective clinical, patient safety, or operational outcomes. Hospitals implementing EHR systems should pair technical deployment with continued training, responsive user support, and systematic evaluation of workflow needs.

## Appendices

Appendix 1: Item-level descriptive statistics

All 20 substantive items used a five-point Likert response format (1 = Strongly disagree to 5 = Strongly agree). Item-level descriptive statistics are reported below, grouped by the four constructs of the analytic framework.

**Table 6:** Item-level descriptive statistics for the 20 substantive Likert items. Note. n = 328. Data are presented as mean (M), standard deviation (SD), minimum (Min), maximum (Max), skewness, and kurtosis. Skewness and kurtosis are Fisher’s adjusted statistics (G1 and excess G2). Composite-level descriptive statistics are reported in Table 2 of the main manuscript.

Item	M	SD	Min	Max	Skewness	Kurtosis
Perceived EHR system success (independent variable; 6 items)						
I use the EHR system regularly in my daily work	4.27	1.22	1	5	-1.57	+1.27
The EHR system is easy to use	3.94	1.11	1	5	-0.87	+0.01
The EHR system supports my clinical or administrative tasks	4.09	1.14	1	5	-1.08	+0.17
The information documented in the EHR system is accurate	4.08	0.97	1	5	-1.07	+1.04
The EHR system allows timely access to patient information	4.29	1.08	1	5	-1.75	+2.53
The EHR system is well integrated across hospital departments	4.00	1.23	1	5	-1.11	+0.27
Perceived healthcare quality and patient safety (DV1; 5 items)						
The EHR system helps reduce documentation errors	4.05	1.12	1	5	-1.13	+0.54
The EHR system improves continuity of patient care	4.30	1.09	1	5	-1.71	+2.24
The EHR system enhances patient safety	4.34	1.06	1	5	-1.93	+3.31
The EHR system improves communication among healthcare staff	4.21	1.05	1	5	-1.50	+1.93
The EHR system supports adherence to clinical guidelines	4.06	1.20	1	5	-1.27	+0.63
Perceived operational efficiency (DV2; 5 items)						
The EHR system reduces duplication of tests and procedures	4.09	1.22	1	5	-1.29	+0.63
The EHR system saves time during daily work activities	3.89	1.33	1	5	-0.96	-0.32
The EHR system improves workflow efficiency	3.97	1.27	1	5	-1.06	-0.01
The EHR system supports better use of hospital resources	4.13	1.08	1	5	-1.14	+0.53
The EHR system contributes to improved operational cost efficiency	4.07	1.10	1	5	-1.19	+0.83
Training and technical support (moderator; 4 items)						
I received adequate training on how to use the EHR system	3.64	1.20	1	5	-0.50	-0.70
The training provided prepared me to use the EHR system effectively	3.69	1.19	1	5	-0.61	-0.43
Technical support is available when I experience EHR problems	3.53	1.30	1	5	-0.54	-0.84
Continuous training improves effective use of the EHR system	4.08	1.11	1	5	-1.02	+0.07

Appendix 2: Sensitivity analyses

Three sensitivity analyses were conducted. The first excluded 48 respondents (14.6% of the full sample) with invariant responses across all 20 substantive items, yielding 280 respondents (85.4% of the full sample), and refitted both hierarchical regression models using the same covariates, main effects, and interaction term. The second refitted the full-sample models after a reflected-log transformation of each negatively skewed outcome using -ln(6 - y). The third entered age group, years of clinical experience, and years using an EHR as categorical indicator variables, with the lowest category as the reference, instead of entering them as ordinal terms.

**Table 7:** Final block focal coefficients in the full sample and invariant-response sensitivity sample. Note. Full sample = 328 participants; sensitivity sample = 280 participants (85.4% of the full sample) after excluding 48 participants (14.6%) with invariant responses. A p-value < 0.05 was considered statistically significant. B = unstandardized coefficient; SE = standard error; β = standardized coefficient; p = two-sided p-value

Outcome/Predictor	Full sample (n = 328)				Sensitivity sample (n = 280)			
	B	SE	β	P-value	B	SE	β	P-value
Perceived healthcare quality and patient safety								
Perceived EHR system success (centered)	+0.771	0.056	+0.711	<0.001	+0.755	0.065	+0.674	<0.001
Training (centered)	+0.078	0.046	+0.078	0.093	+0.067	0.054	+0.063	0.221
Perceived EHR system success × training	−0.048	0.034	−0.057	0.162	−0.082	0.057	−0.074	0.148
Perceived operational efficiency								
Perceived EHR system success (centered)	+0.668	0.069	+0.571	<0.001	+0.644	0.079	+0.530	<0.001
Training (centered)	+0.214	0.056	+0.199	<0.001	+0.202	0.066	+0.177	0.003
Perceived EHR system success × training	−0.043	0.042	−0.047	0.309	−0.101	0.069	−0.083	0.145

**Table 8:** Standardized focal coefficients in primary and reflected-log outcome models. Note. n = 328. β = standardized coefficient; p = two-sided p value. Reflected-log outcomes were calculated as −ln(6 − y). A p-value < 0.05 was considered statistically significant. The association of training and technical support with perceived healthcare quality and patient safety changed from nonsignificant in the primary model to significant in the transformed model; this estimate is therefore sensitive to outcome specification.

Outcome/Predictor	Primary β	Primary p	Reflected-log β	Reflected-log p
Perceived healthcare quality and patient safety				
Perceived EHR system success (centered)	0.711	<0.001	0.705	<0.001
Training and technical support (centered)	0.078	0.093	0.162	<0.001
EHR success × training/support	−0.057	0.162	0.059	0.148
Perceived operational efficiency				
Perceived EHR system success (centered)	0.571	<0.001	0.571	<0.001
Training and technical support (centered)	0.199	<0.001	0.251	<0.001
EHR success × training/support	−0.047	0.309	0.057	0.229

**Table 9:** Focal coefficients from the sensitivity models using categorical indicator coding. Note. n = 328. Age group, years of clinical experience, and years using an EHR were entered as categorical indicator variables, with the lowest category as the reference. All other model terms were unchanged. A p-value < 0.05 was considered statistically significant. B = unstandardized coefficient; SE = standard error; β = standardized coefficient; p = two-sided p-value

Outcome/Predictor	B	SE	β	P-value
Perceived healthcare quality and patient safety				
Perceived EHR system success (centered)	0.769	0.057	0.709	<0.001
Training and technical support (centered)	0.078	0.047	0.079	0.095
EHR success × training/support	-0.049	0.035	-0.059	0.154
Perceived Operational Efficiency				
Perceived EHR system success (centered)	0.661	0.069	0.565	<0.001
Training and technical support (centered)	0.212	0.057	0.198	<0.001
EHR success × training/support	-0.046	0.042	-0.051	0.271
